# PTHrP Overexpression Increases Sensitivity of Breast Cancer Cells to Apo2L/TRAIL

**DOI:** 10.1371/journal.pone.0066343

**Published:** 2013-06-18

**Authors:** Vanessa Cheung, Steve Bouralexis, Matthew T. Gillespie

**Affiliations:** 1 Bone, Joint and Cancer Unit, Prince Henry’s Institute, Melbourne, Victoria Australia; 2 Department of Biochemistry and Molecular Biology, Monash University, Melbourne, Victoria Australia; The University of Kansas Medical center, United States of America

## Abstract

Parathyroid hormone-related protein (PTHrP) is a key component in breast development and breast tumour biology. PTHrP has been discovered as a causative agent of hypercalcaemia of malignancy and is also one of the main factors implicated in breast cancer mediated osteolysis. Clinical studies have determined that PTHrP expression by primary breast cancers was an independent predictor of improved prognosis. Furthermore, PTHrP has been demonstrated to cause tumour cell death both *in vitro* and *in vivo*. Apo2L/TRAIL is a promising new anti-cancer agent, due to its ability to selectively induce apoptosis in cancer cells whilst sparing most normal cells. However, some cancer cells are resistant to Apo2L/TRAIL-induced apoptosis thus limiting its therapeutic efficacy. The effects of PTHrP on cell death signalling pathways initiated by Apo2L/TRAIL were investigated in breast cancer cells. Expression of PTHrP in Apo2L/TRAIL resistant cell line MCF-7 sensitised these cells to Apo2L/TRAIL-induced apoptosis. The actions of PTHrP resulted from intracellular effects, since exogenous treatment of PTHrP had no effect on Apo2L/TRAIL-induced apoptosis. Apo2L/TRAIL-induced apoptosis in PTHrP expressing cells occurred through the activation of caspase-10 resulting in caspase-9 activation and induction of apoptosis through the effector caspases, caspase-6 and -7. PTHrP increased cell surface expression of Apo2L/TRAIL death receptors, TRAIL-R1 and TRAIL-R2. Antagonistic antibodies against the death receptors demonstrated that Apo2L/TRAIL mediated its apoptotic signals through activation of the TRAIL-R2 in PTHrP expressing breast cancer cells. These studies reveal a novel role for PTHrP with Apo2L/TRAIL that maybe important for future diagnosis and treatment of breast cancer.

## Introduction

Breast cancer is one of the highest causes of cancer related deaths amongst women. Despite advances in the detection of localised disease and a decline in the mortality rates of primary breast cancer patients, current therapies are only palliative for advanced metastatic breast cancer patients. Approximately 70% of women with advanced breast cancer will have bone metastases [1]. Once tumour cells metastasise to bone, mortality increases to 70% [2]. Thus a greater understanding of tumour progression and the key factors involved is vital not only for understanding cancer biology but also for improving cancer treatment.

Parathyroid hormone-related protein (PTHrP) was discovered as the causative agent of hypercalcaemia in cancer patients [3]. Since its discovery the involvement of PTHrP in the hypercalcaemia of breast cancer has been extensively studied. PTHrP has also been implicated in breast cancer progression and the bone metastasis process [4], [5]. In the bone microenvironment, PTHrP is involved in the osteotrophism of breast cancer cells, through its ability to activate osteoclastic bone resorption and thus participation in driving the ‘vicious cycle’ [4]. Studies showed that PTHrP levels were much higher in primary tumours of breast cancer patients who later developed bone metastasis [6]–[8], thus leading to the hypothesis that PTHrP expression in primary breast tumours increases the probability of bone metastasis and decreased patient survival. Contrary to this, a larger clinical study that examined the relationship between PTHrP production and bone metastasis in patients with operable breast cancer revealed that patients with PTHrP positive tumours had significantly improved survival rate with less metastases to bone than patients with PTHrP-negative tumours [5], [9]. Together, these studies support the idea of a dual role for PTHrP in breast cancer, a protective function early on in the disease leading to improved survival and reduced metastasis, and a destructive role once the tumour progresses and metastasise to the bone.

Apo2 ligand (Apo2L/TRAIL) is a member of the tumour necrosis factor (TNF)-cytokine family that can induce apoptosis in a variety of transformed cells, including breast cancer, whilst sparing most non-transformed cells [10]–[12]. Apo2L/TRAIL is a type II transmembrane protein that induces apoptosis through interactions with its death receptors; TRAIL-R1/DR4 and TRAIL-R2/DR5 [13], [14]. Recombinant Apo2L/TRAIL and agonistic antibodies targeting Apo2L/TRAIL receptors are currently in clinical trials for cancer. Mapatumumab, an agonistic antibody against TRAIL-R1, is in Phase II clinical trials in patients with colorectal cancer and non-small cell lung cancer [15], [16]. However, one of the main hurdles of Apo2L/TRAIL therapy is that many cancer cells remain resistant to Apo2L/TRAIL-induced apoptosis. Although many methods have been identified to overcome Apo2L/TRAIL resistance such as combination therapy with chemotherapeutics and other biological reagents, the mechanism of Apo2L/TRAIL sensitivity and/or resistance and strategies to overcome drug resistance still remains to be explored.

In this study, we demonstrate that PTHrP expression in breast cancer cells sensitised them to Apo2L/TRAIL, and in deed converted MCF-7 cells from Apo2L/TRAIL resistant cells to respond to Apo2L/TRAIL-induced apoptosis. Apo2L/TRAIL induced apoptosis in PTHrP overexpressing cells through the activation of caspase-10 resulting in caspase-9 activation and induction of apoptosis through the effector caspases; caspase-6 and -7. There was an increase in cell surface expression of TRAIL-R1 and TRAIL-R2 in PTHrP overexpressing cells. Antagonistic antibodies against the death receptors demonstrated that Apo2L/TRAIL preferentially bound to TRAIL-R2 to promote apoptosis in the PTHrP overexpressing cells.

## Materials and Methods

### Cells and Reagents

MCF-7 and MDA-MB-231 human breast carcinoma cell lines were obtained from the American Type Culture Collection (Rockville, MD). Cells were cultured in RPMI 1640, supplemented with 10% foetal bovine serum, glutamine (2 mM), HEPES (16.8 mM) penicillin-streptomycin (10,000 U/ml) and minocyclin (1 mg/L) (Life Technologies, Inc.), in a humidified atmosphere containing 5% CO_2_. MCF-7 PTHrP overexpressing cells are as previously described [17]. Human recombinant Apo2L/TRAIL was obtained from Preprotech (USA). Monoclonal antibodies against human Apo2L/TRAIL-R1/DR4 (MAB347), Apo2L/TRAIL-R2/DR5 (MAB6311), Apo2L/TRAIL-R3/DcR1 (MAB6302), Apo2L/TRAIL-R4 (MAB633), goat polyclonal antibodies against human Apo2L/TRAIL-R1/DR4 (AF347), Apo2L/TRAIL-R2/DR5 (AF631), Apo2L/TRAIL-R3/DcR1 (AF630), Apo2L/TRAIL-R4 (AF633), mouse IgG_2B_ (MAB004), mouse IgG_1_ (MAB002)_,_ monoclonal antihuman Caspase-7 (MAB823), Caspase-8 (MAB704), Caspase-9 (MAB8301), Caspase-10 (MAB834) were from R&D Systems (MN, USA). Polyclonal antihuman Caspase-6 (9762) was purchased from Cell Signalling Technology (MA, USA). Monoclonal β-actin antibody (A5316) was from Sigma-Aldrich (MO, USA). Monoclonal PARP antibody (C2–10) (4338-MC-50) was from Trevigen (MD, USA). Goat anti-mouse HRP was from Cell Signalling (MA, USA). Goat anti-rabbit-HRP (P0448) was from Dako Cytomation (Denmark).

### Measurement of Cell Viability

For determination of Apo2L/TRAIL mediated cytotoxicity, 1×10^4^ cells per well were seeded into 96-well microtiter plates and allowed to adhere to the plate for 24 h. Cells were treated according and/or then treated with 100 ng/ml Apo2L/TRAIL for 24 h. Cell viability was determined by staining the cells with crystal violet and measuring the OD_570_ of cell lysates.

DAPI staining of nuclei- Cells were seeded on plastic chamber slides and stimulated as indicated. After 2 washes with PBS, cells were fixed in methanol for 5 min, washed again with PBS and incubated with 0.8 mg/ml of 4′, 6-diamidine-2′-phenylinodole dihydrochloride (DAPI, Roche Diagnostics, Castle Hill, NSW, Australia) in PBS for 15 min at 37°C. After several washes in PBS, the coverslips were mounted on PBS/glycerine. DAPI was visualised by fluorescence microscopy.

### Western Blot

Cells were treated as indicated and lysed in buffer containing; 50 mM Tris HCL, 150 mM NaCl, 1% NP-40, 0.5% sodium deoxycholate, 0.1% SDS and protease cocktail inhibitor (1/100) and scraped. After centrifugation at 13,000 rpm at 4°C for 10 min, the total protein concentration in the extracts were determined using BCA protein assay (Thermo Fisher Pierce). Proteins were resolved by Bis-Tris gel electrophoresis (Life Technologies) and transferred to PVDF membranes (Millipore). Membranes were blocked in 5% skim milk powder in 1xPBS and were successively incubated with the following antibodies: anti-human caspase -6, -7, -8, -9, -10 and PARP, anti-human β-tubulin (Roche) and peroxidise-conjugated goat anti-mouse IgG (Cell Signalling). Peroxidase activity was revealed using an enhanced chemiluminescence kit (Roche).

### Flow Cytometry

Cells were cultured accordingly then washed twice with PBS and detached using 2 mM EDTA in PBS at 37°C for 5 min. For flow cytometric analysis, all subsequent incubation steps were performed on ice and centrifugation steps performed at 4°C. For analysis of Apo2L/TRAIL receptor expression, cells were resuspended in ice cold PBS and centrifuged for 6 min at 12,000 rpm. Cells were resuspended in ice cold PBS and PBS/Azide solution (0.1% Azide) and centrifuged at 12,000 rpm, 5 min. Cells were resuspended at 2×10^6^ cells/ml in blocking buffer (10% BSA/PBS +0.1% Azide). Monoclonal antibodies or the isotype-matched nonbinding control antibodies were diluted in blocking buffer to 10 µg/ml, added to 50 µl aliquots of cell suspension and incubated for 45 min. Cells were then washed twice with 2 ml of wash buffer and collected by centrifugation. PE-conjugated antibody was added to the resuspended cell pellets, diluted 1/50 in wash buffer. The cells were incubated for a further 45 min in the dark, washed three times as above, then resuspended and fixed in 0.3 ml ice-cold 1% w/v paraformaldehyde for analysis. For analysis of cell cycle, cells were cultured for 24 h, then serum starved for a further 24 h and media was replaced with growth media. At the appropriate time point cells were detached as above and collected by centrifugation. Cells were washed in ice cold PBS and centrifuged. Cells were then resuspended in 200 µl PBS +0.1% FBS, and fixed in ice cold 70% ethanol then incubated for 1 h at 4°C. Cells were washed as above and resuspended in 1 ml of solution containing; 0.1% Triton-X 100, 200 µg/ml RNAse free and 40 µg/ml propidium iodide, incubated for 20 min at 37°C then analysed.

### Apoptotic DNA Laddering Assay

Cells were cultured and treated with 100 ng/ml recombinant Apo2L/TRAIL or left untreated with 24 h. DNA was isolated and treated using the Apoptotic DNA Ladder Kit (Roche, Germany) according to the manufacture’s protocol.

### Cell Growth Assays

Cells were seeded at a density of 6×10^4^ cells per well in a 6 well plate and allowed to adhere for 24 h. Cells were serum starved for 24 h by replacing media with serum free media. After 24 h cells were released with replacement of full serum media. Cells were collected at various time points by the addition of trypsin and resuspended in PBS prior to counting.

### Statistical Analysis

All data are presented as the mean ± standard deviation (SD) unless stated otherwise. Statistically significant differences were determined by unpaired t-test or one-way ANOVA followed by Turkeys post hoc test to identify pair wise differences. In all cases, p<0.05 was considered significant. Statistical analyses were carried out using MS Excel 2003 (CA, USA) or GraphPad Prism 5 (CA, USA).

## Results

### PTHrP Overexpression Increases Cell Growth of Breast Cancer Cells

**Figure 1:**
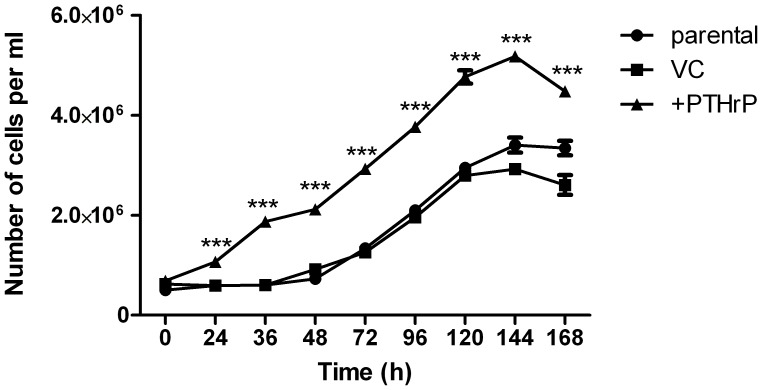
PTHrP increases growth of breast cancer cells. Cells (6×10^4^ per well) were seeded in triplicate into 6 well plates and allowed to adhere for 24 h. Cells were serum starved for 24 h then media was replaced with full serum media. Cell growth was determined by collecting cells every 24 h for a total of 7 days and counted using a Coulter counter. Data is a representative of three independent experiments, the mean ± SD of 6 replicates are provided. ***p<0.001 relative to parental or vector control (VC) cells.

To assess the effects of PTHrP in breast cancer cells, PTHrP (1–139 aa) was overexpressed in MCF-7 cells and clonal cells were generated from positively transfected cells, and these cells have been previously described [17]. Both mRNA and protein expression was validated for PTHrP expression in these transfected cells: the PTHrP overexpressing cells produce PTHrP at 1609±171 pmol/liter/10^5^ cells compared with parental and vector control cells which express PTHrP at 1.8±0.4 pmol/liter and 4.3±0.3 pmol/liter/10^5^ cells, respectively [17]. The effect of PTHrP overexpression upon cell proliferation was assessed by counting cell growth over a period of 7 days (**Fig. 1**). Cells were seeded into 6 well plates and allowed to adhere for 24 h. The cells were then synchronised with serum free media for 24 h, then released with the replacement of full serum media. MCF-7 cells overexpressing PTHrP attained a higher number of cells from 24 h and throughout the rest of the culture period, when compared to MCF-7 parental or MCF-7 vector control cells (**Fig. 1**).

**Figure 2:**
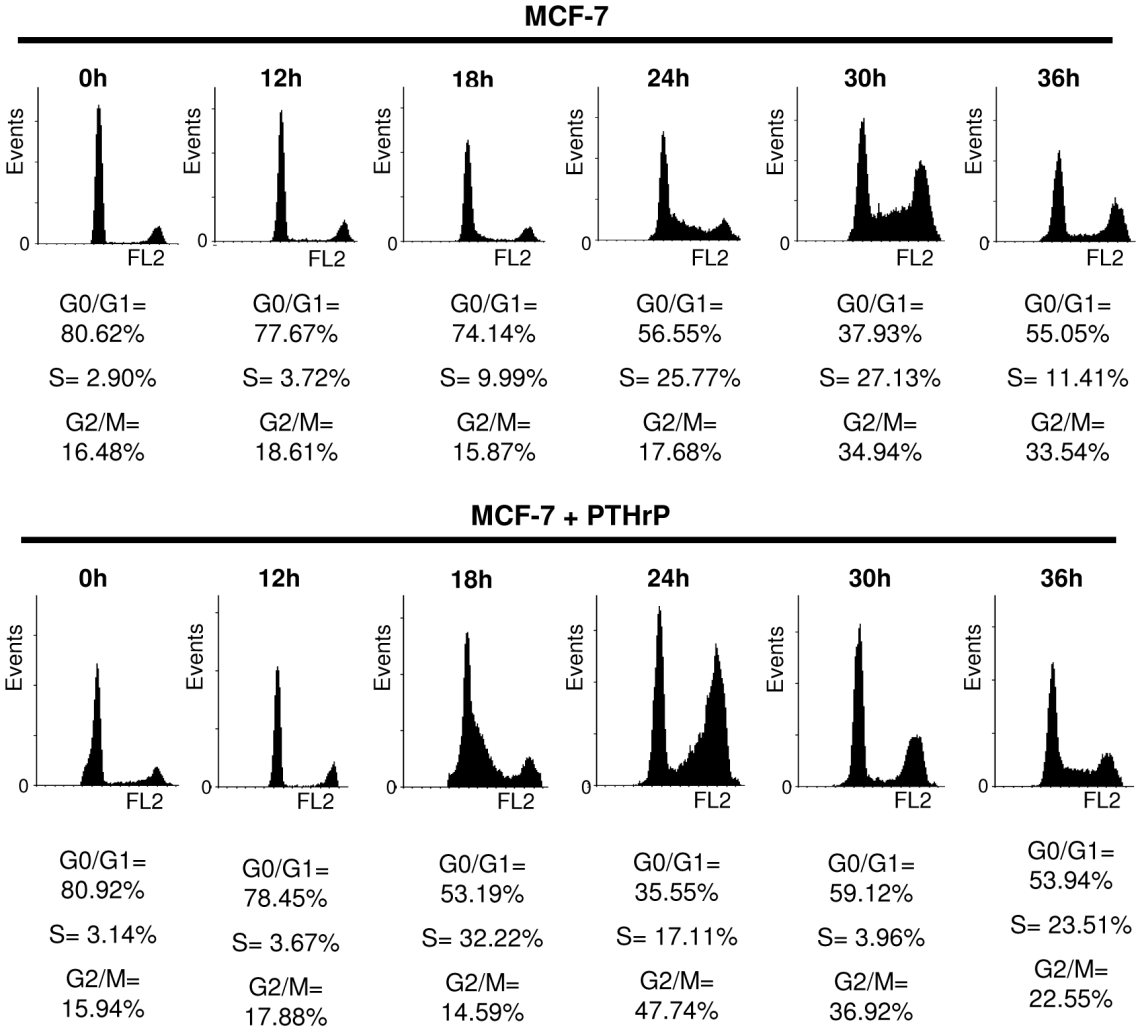
PTHrP increases cell cycle progression of breast cancer cells. Cells were serum starved for 24 h then media was replaced with full serum media. Cells (**A**, MCF-7 and **B**, MCF-7+PTHrP) were fixed in 70% ethanol, washed then stained with a solution containing 40 µg/ml propidium iodide and analysed by flow cytometric analysis. Data presented is a representative of three independent experiments.

Since greater cell numbers were observed by cells overexpressing PTHrP, FACS analyses was performed to determine the rate of cell cycle progression in response to PTHrP overexpression. Cell cycle was analysed by staining cellular DNA content with propidium iodide and reading fluorescent cells by flow cytometry. Cells were synchronised and released with replacement of full serum media. Cells were then fixed, stained and analysed at various time points over 24 h. The FACS histograms revealed that PTHrP overexpressing cells entered into the S phase earlier (32% of PTHrP overexpressing cells compared with 10% of parental cells) (**Fig. 2**). The PTHrP overexpressing cells entered into a second cycle of the S phase with 23% of cells at 36 h (**Fig. 2**). The increase in cell cycle progression may account for the increase in cell numbers. These results confirm data that has already been described where PTHrP overexpression in breast cancer cells enhances cell cycle progression [18].

### Expression of PTHrP Sensitises MCF-7 Cells to Apo2L/TRAIL-induced Apoptosis

**Figure 3:**
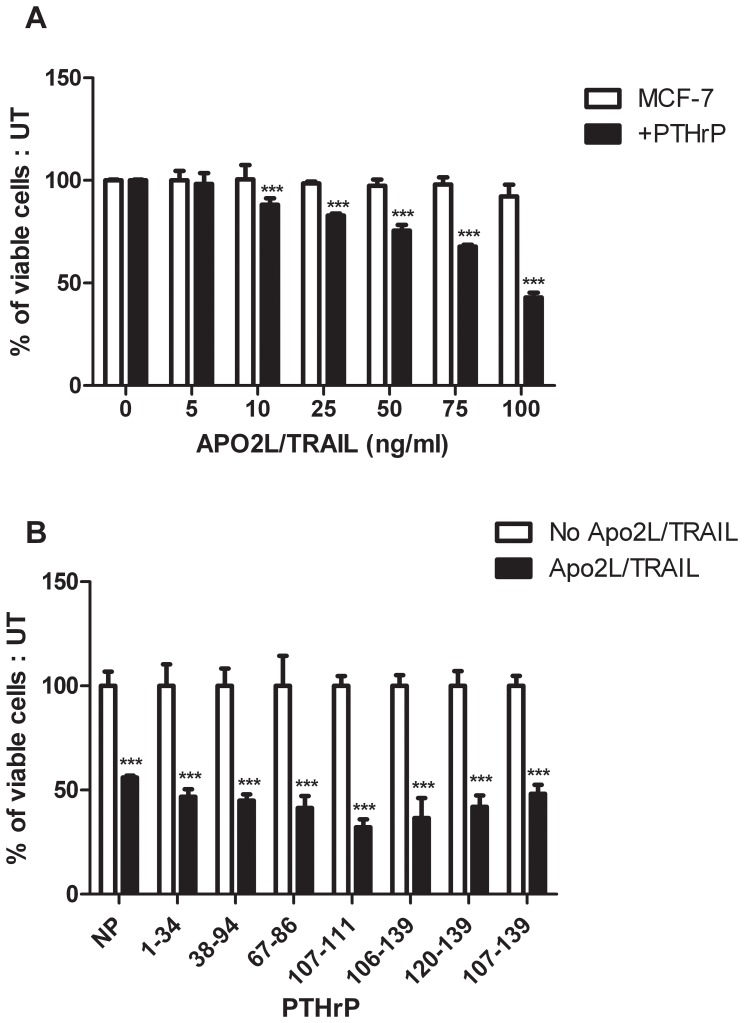
PTHrP sensitises breast cancer cells to Apo2L/TRAIL induced cell death. **A**) MCF-7 and MCF-7+PTHrP cells (1×10^4^ cells per well) were seeded in quadruplicate into 96 well plates and treated with Apo2L/TRAIL or untreated for 24 h. Cell viability was determined by crystal violet staining and quantified by OD_570_ measurement using a spectrophotometer. Data are presented as the mean ± SD of three independent experiments and are expressed as a percentage of the untreated control cells. **B**) MDA-MB-231 cells were cultured as above and treated with various portions of PTHrP (10^−8^ M) for 12 h, followed by treatment with Apo2L/TRAIL for a further 12 h. Cell viability was assessed as above. ***p<0.001 relative to untreated controls.

Since PTHrP overexpression has been shown to alter cellular growth and affect apoptosis [19], [20], the role of PTHrP in cell death was assessed in breast cancer cells treated with Apo2L/TRAIL. Breast cancer cells were cultured for 24 h then treated with increasing doses of Apo2L/TRAIL for 24 h. Cell death was not noted in MCF-7 cells even with maximal treatment dose of 100 ng/ml of Apo2L/TRAIL for 24 h. In contrast, MCF-7 PTHrP overexpressing cells displayed a decrease in cell viability with an increase in dose of Apo2L/TRAIL treatment. Maximal cell death of 50% was observed with a treatment dose of 100 ng/ml of Apo2L/TRAIL when compared to untreated cells, and a dose of 10 ng/ml Apo2L/TRAIL provided a significant difference in cell death relative to untreated cells (**Fig. 3A**).

In view of the results above, the effects of various portions regions of PTHrP were assessed to determine whether the increase in sensitivity to Apo2L/TRAIL treatment was the result of intracellular or extracellular actions of PTHrP. MDA-MB-231 cells were utilised as they are sensitive to Apo2L/TRAIL treatment and as they 1) have higher levels of endogenous PTHrP levels and 2) higher PTH1R expression compared with MCF-7 cells. MDA-MB-231 cells were treated with PTHrP 1–34 aa peptide, which activates PTH1R [21], for 12 h then treated with 100 ng/ml Apo2L/TRAIL for a further 12 h. Treatment with Apo2L/TRAIL confirmed MDA-MB-231 sensitivity to Apo2L/TRAIL induced cell death with 56% viable cells (**Fig. 3B**). Treatment with PTHrP 1–34 aa peptide did not show a significant decrease when compared to Apo2L/TRAIL treatment alone (**Fig. 3B**). Similarly, treatment of MDA-MB-231 cells with PTHrP fragments 38–94 aa, 67–86 aa, 106–139 aa, 120–139 aa, 107–111 aa and 107–139 aa did not result in a significant alteration in the percentage of viable cells when treated with Apo2L/TRAIL, when compared to Apo2L/TRAIL treatment alone (**Fig. 3B**). The results suggest that increased Apo2L/TRAIL sensitivity is not the result of extracellular receptor-mediated actions of PTHrP, and were more likely to result from intracellular actions of PTHrP. We also investigated the actions of TRAIL on an MDA-MB-231 cell line in which PTHrP expression had been knocked-down by an anti-sense approach. In this cell line only a 50% reduction of PTHrP expression (mRNA and protein) was determined and no significant difference in TRAIL effects was noted between the knock-down cells when compared with their parental cells; percentage of viable cells 52.5% and 59.3%, respectively (data not shown). This suggests that the magnitude of suppression of PTHrP expression was insufficient to lessen the sensitivity of these cells to the effects of TRAIL.

### PTHrP Overexpression Sensitised MCF-7 Cells to Apo2L/TRAIL-induced Apoptosis via Activation of the Intracellular Apoptotic Pathway

**Figure 4:**
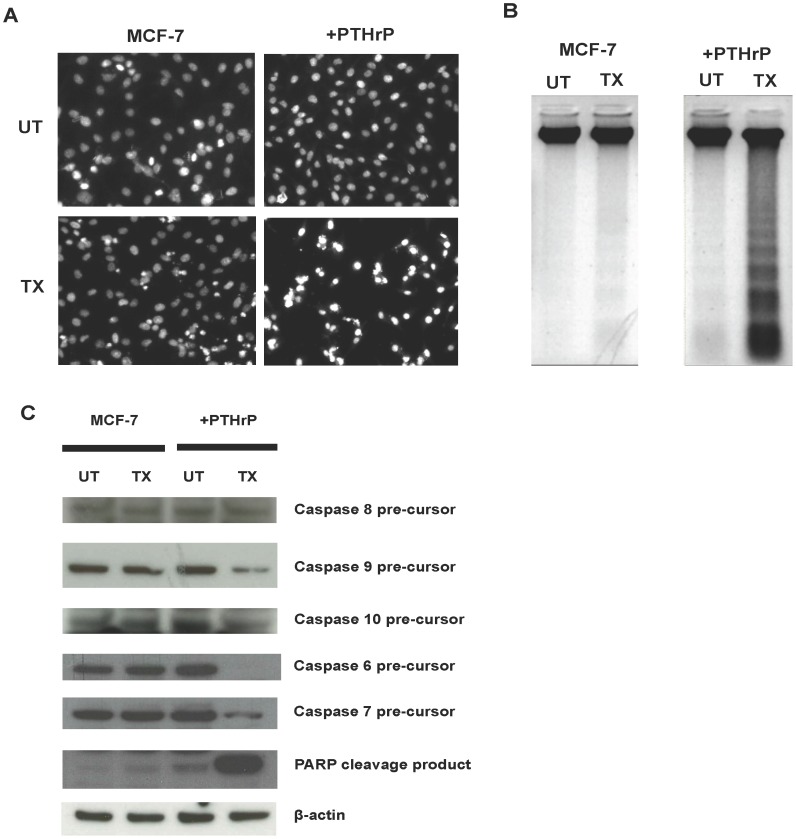
Apo2L/TRAIL induces apoptosis in PTHrP overexpressing breast cancer cell through activation of the intracellular apoptotic pathway. **A**) Cells were seeded onto chamber slides and treated with Apo2L/TRAIL (TX) or untreated (UT) for 24 h. Cells were fixed with methanol and incubated with DAPI, before washing in PBS and mounting with PBS/glycerine. DAPI staining was visualised by fluorescence microscopy. **B**) Apoptosis was assessed by DNA laddering assay. Cells were cultured and treated with Apo2L/TRAIL (TX) or untreated (UT) for 24 h. DNA was isolated and treated using the Apoptotic DNA Laddering Kit (Roche). **C**) Caspase expression. Cells were cultured as indicated above and lysed after 24 h. Cell extracts were analysed by Bis-Tris gel electrophoresis and transferred to PVDF membranes. Caspase pre-cursors-6, -7, -8, -9, -10, PARP and β-actin protein levels was assessed by immunoblotting.

To confirm that the reduction of viable cell number in PTHrP overexpressing breast cancer cells treated with Apo2L/TRAIL was due to an induction of apoptosis, cells were cultured with Apo2L/TRAIL, stained with DAPI and assessed under fluorescent microscopy. Morphological evidence of apoptosis was observed in Apo2L/TRAIL-treated cells including chromatin condensation and nuclear DNA fragmentation (**Fig. 4A**). Apoptotic cells were not observed in MCF-7 cells treated with Apo2L/TRAIL (**Fig. 4A**). A lower number of viable cells in Apo2L/TRAIL treated PTHrP overexpressing cells was observed compared to MCF-7 parental cells (**Fig. 4A**).

Another hallmark of apoptosis is the formation of DNA fragments of oligonucleosomal size ranging from 180 to 200 bp. DNA laddering assays were performed on MCF-7 PTHrP overexpressing cells treated with Apo2L/TRAIL. PTHrP overexpressing cells treated with Apo2L/TRAIL displayed DNA laddering indicative of apoptosis compared to untreated cells (**Fig. 4B**). No discernable DNA laddering was observed with the MCF-7 cells that were untreated or treated with Apo2L/TRAIL (**Fig. 4B**).

To examine the molecular mechanism of Apo2L/TRAIL-induced apoptosis in PTHrP overexpressing breast cancer cells, the expression and processing of intracellular proteins involved in the intrinsic apoptotic signalling pathway was assessed by immunoblotting. MCF-7 cells contain a mutation in caspase-3 rendering it inactive, thus these cells cannot activate apoptosis via the extrinsic pathway. In both the MCF-7 and PTHrP overexpressing cells, Apo2L/TRAIL treatment did not alter the levels of caspase-8 precursor protein compared to untreated controls (**Fig. 4C**). Treatment of PTHrP overexpressing cells with Apo2L/TRAIL decreased levels of precursor proteins for caspase-9, -10, -6 and -7 (**Fig. 4C**). There was no change in precursor caspase-10, -9, -6 and -7 levels in Apo2L/TRAIL treated MCF-7 cells compared to untreated controls (**Fig. 4C**). These results indicate that these caspases are activated in PTHrP overexpressing cells treated with Apo2L/TRAIL. Activated caspases can cleave PARP, which then facilitates the cell death process and thus can serve as an apoptosis marker. Consistent with this notion, elevated levels of PARP cleavage product was observed in PTHrP overexpressing cells treated with Apo2L/TRAIL compared to untreated control cells and treated MCF-7 parental cells (**Fig. 4C**).

### Cell Surface Expression of TRAIL-R1 and TRAIL-R2 was Elevated in PTHrP-overexpressing Cells

**Figure 5:**
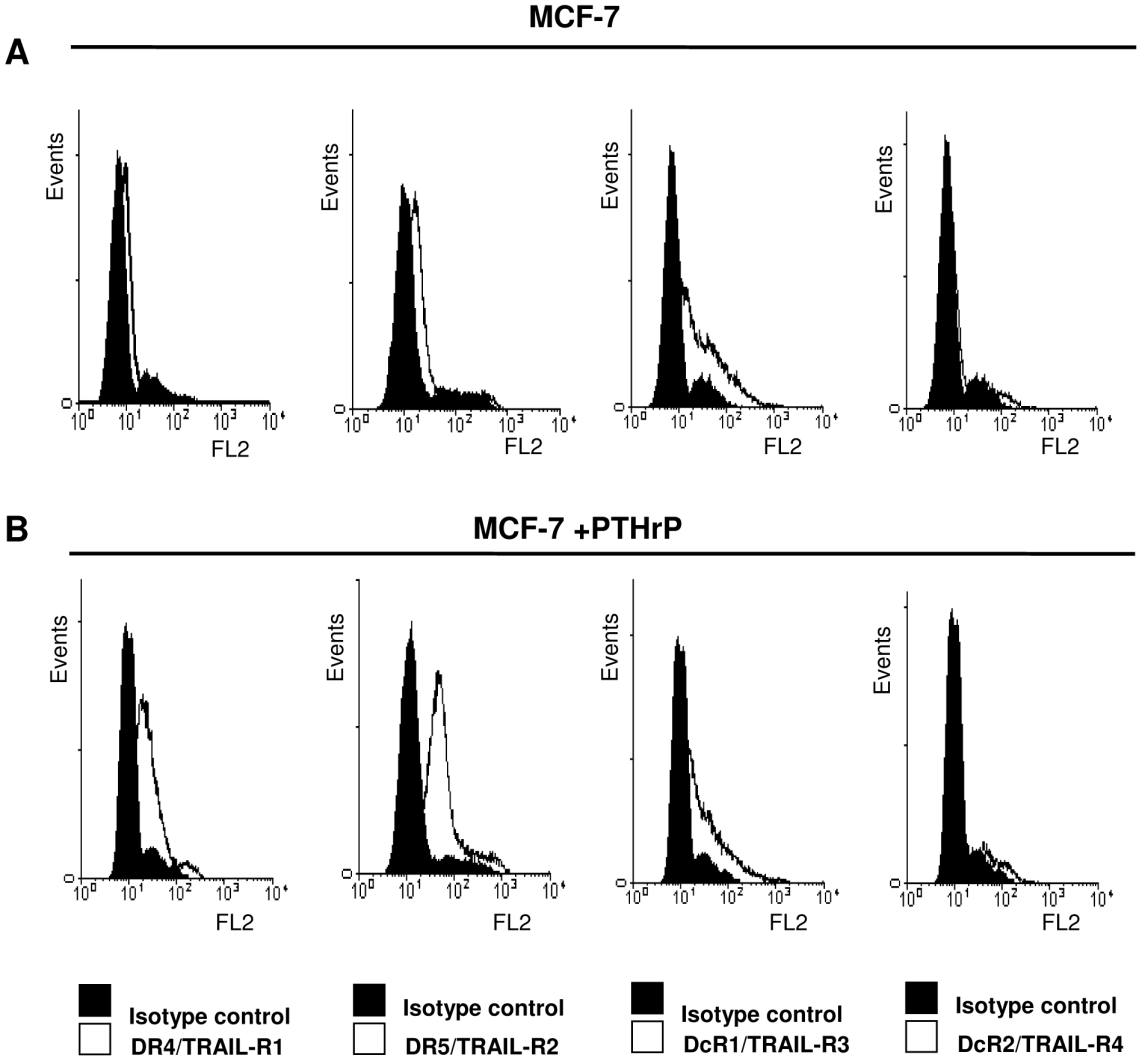
The cell surface expression of TRAIL-R1 and TRAIL-R2 is induced in PTHrP overexpressing cells. Cells (**A**, MCF-7 and **B**, MCF-7+PTHrP) were cultured, washed in PBS and blocked in 10% BSA/PBS +0.1% Azide. Monoclonal antibodies against specific for the human Apo2L/TRAIL death and decoy receptors were added to the cells, and were detected by PE-conjugated secondary antibodies. Cells were washed in PBS and fixed in 1% paraformaldehyde before detection of receptors by flow cytometric analysis. Data presented is a representative of three independent experiments.

To determine whether there was any correlation between the sensitisation of cells to Apo2L/TRAIL and the levels of expression of Apo2L/TRAIL receptors, immunofluorescent staining of each of the Apo2L/TRAIL receptors was performed and analysed using flow cytometry (**Fig. 5A, B**). The cell surface expression levels of TRAIL-R1 and TRAIL-R2 were elevated in the Apo2L/TRAIL sensitive cell line, MCF-7 PTHrP overexpressing cells compared to MCF-7 parental cells (**Fig. 5B**). The expression levels of both the decoy receptors, TRAIL-R3 and TRAIL-R4, were low and equivalent in both the MCF-7 parental and PTHrP overexpressing cells (**Fig. 5A, B**).

### Inhibition of TRAIL-R2 Protects against Apo2L/TRAIL-induced Apoptosis in PTHrP-overexpressing Cells

**Figure 6:**
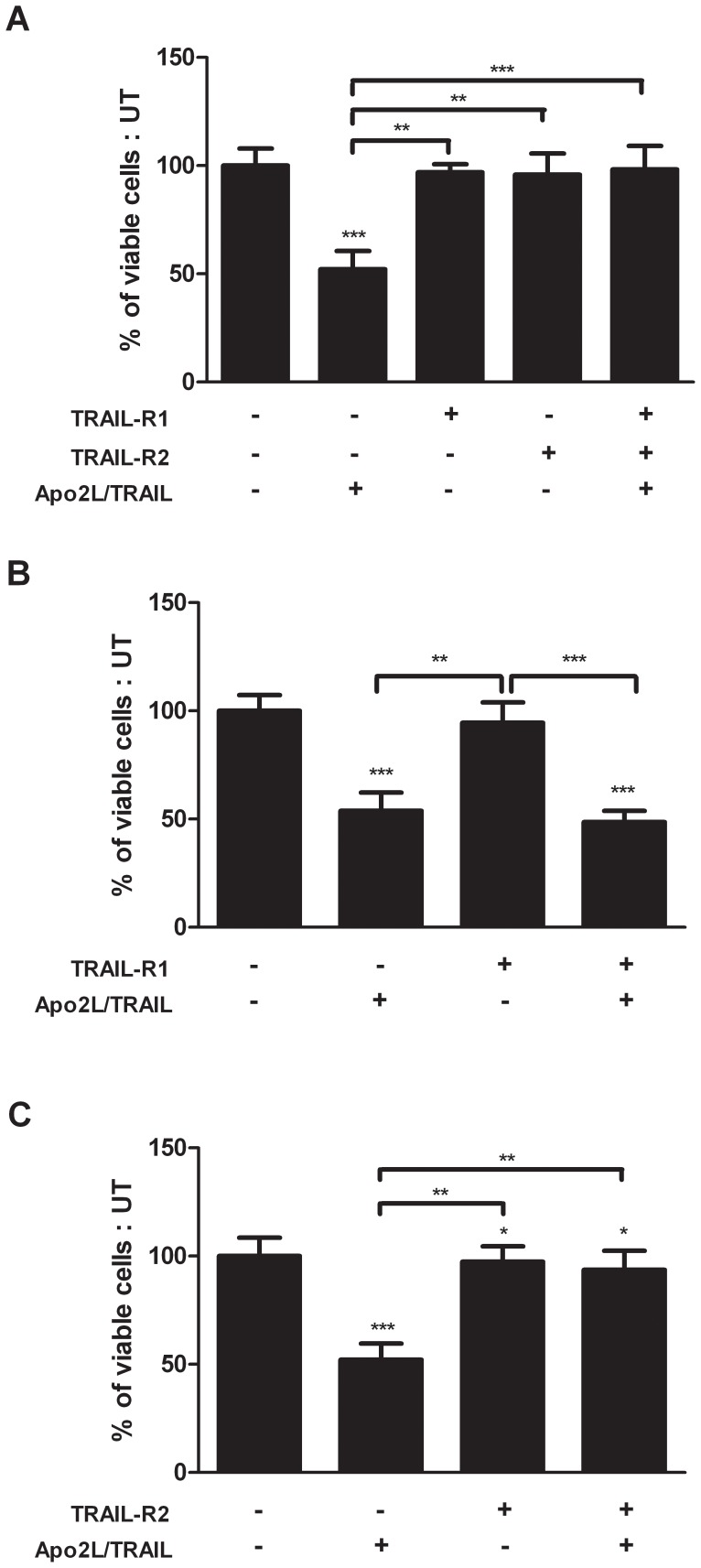
Apo2L/TRAIL induced apoptosis in PTHrP overexpressing breast cancer cells is signalled through TRAIL-R1. Cells were cultured and incubated with polyclonal antibodies against **A**) TRAIL-R1 and TRAIL-R2, **B**) TRAIL-R1 only and **C**) TRAIL-R2 only for 24 h, then treated with Apo2L/TRAIL for a further 24 h. Cell viability was assessed by crystal violet staining and quantified by OD_570_ measurement using a spectrophotometer. Data are presented as the mean ± SD of three independent experiments and are expressed as a percentage of the untreated control cells, *p<0.05, **p<0.01, ***p<0.001.

To establish whether Apo2L/TRAIL treatment was associated with a functional activation of TRAIL-R1 or TRAIL-R2, MCF-7 PTHrP overexpressing cells were treated with antagonistic anti-TRAIL-R1 and anti-TRAIL-R2 antibodies (10 µg/ml) followed by 100 ng/ml Apo2L/TRAIL and cell survival was assessed. Treatment with Apo2L/TRAIL alone induced 50% cell death when compared to untreated cells (**Fig. 6A**). When cells were pre-treated with both antagonistic antibodies against TRAIL-R1 and TRAIL-R2 no cell death was observed, and was similar to that of untreated cells (**Fig. 6A**). To elucidate which of the two Apo2L/TRAIL death receptors were used for Apo2L/TRAIL signalling, each antagonistic antibody was used individually. When the cells were pre-incubation with anti-TRAIL-R1 followed by Apo2L/TRAIL treatment, 50% cellular apoptosis was observed which was similar to the levels detected with Apo2L/TRAIL treatment alone (**Fig. 6B**). Pre-incubation with anti-TRAIL-R2 followed by Apo2L/TRAIL treatment inhibited Apo2L/TRAIL-induced apoptosis, with the percentage of viable cells similar to the untreated controls or treatment with anti-TRAIL-R2 alone (**Fig. 6C**). Combined, these results indicate that Apo2L/TRAIL is signalling via binding to and activation of TRAIL-R2, and not TRAIL-R1, to induce the apoptosis signal in MCF-7 PTHrP overexpressing cells.

## Discussion

PTHrP was originally identified as a tumour factor responsible for HHM, since then it has been well established that PTHrP is expressed in a wide variety of tissues in the foetus and adult (reviewed in [22], [23]). The predominant roles of PTHrP relate to development, growth, smooth muscle tone and calcium transport (reviewed in [24]). PTHrP also has a significant role in mammary gland development and function. Both overexpression and underexpression of PTHrP has been shown to disrupt branching morphogenesis during mammary gland development. Firstly, PTHrP rescued-knock out mice failed to develop mammary glands [22]. In PTHrP null and PTH1R knockout mice, the mammary epithelial buds form, but there is a complete failure of formation of a duct system and instead the mammary epithelial ducts degenerate and disappear by birth [25]–[27]. In addition, overexpression of PTHrP in mice, displayed impaired ductal proliferation and elongation and branching morphogenesis during mammary development at puberty and in early pregnancy [28].

Many studies have demonstrated a role for PTHrP in cell growth and apoptosis [18]–[20], [29]–[31], and notably the following observations have been made: 1. PTHrP when overexpressed in MCF-7 cells protected cells from serum starvation-induced apoptosis, due to an increase in anti-apoptotic proteins Bcl-2 and Bcl-x_L_ [19]; 2. in HEK293 cells, PTHrP inhibited TNFα-induced apoptosis by blocking the extrinsic and intrinsic pathway through regulation of Bcl-2 family of proteins [30]; 3. mutation of the nuclear localisation signal region of PTHrP ablated both apoptotic and proliferative responses in chondrocytes, whilst extracellular PTHrP had no effect [29]; 4. the mid-region PTHrP (50–86 aa) was able to restrain growth and invasion as well as cause striking toxicity and accelerated death of a panel of breast cancer cell lines, the most responsive being MDA-MB-231 [20]; and 5. that extracellular PTHrP treatment induced the up-regulation of pro-apoptotic genes Bcl-xS, Bad and Rip1 was switches on the expression of caspase -2, -5, -6, -7 and -8 in MDA-MB-231 cells [31]. These studies suggest intracellular actions of PTHrP on apoptosis, and possible regulation of apoptosis through regulation of Bcl-2 family members.

We have previously described that PTHrP has paracrine, autocrine and intracrine actions, and demonstrated that PTHrP can target to the nucleus as a result on intracellular trafficking via importin beta, and that extracellular PTHrP can be internalized as a result of binding to the PTH1R, and that PTHrP can attain different subcellular locations [21], [32]–[35]. With our extensive knowledge into the divergent actions and receptor domains of PTHrP, we used a series of peptides that encapsulate the biologically-active domains of PTHrP, and we have previously demonstrated to be biologically active, to demonstrate that the actions were not the result of extracellular signaling, or internalization of PTHrP peptides, leading to the proposition that these PTHrP actions were the result of intracellular actions.

**Figure 7:**
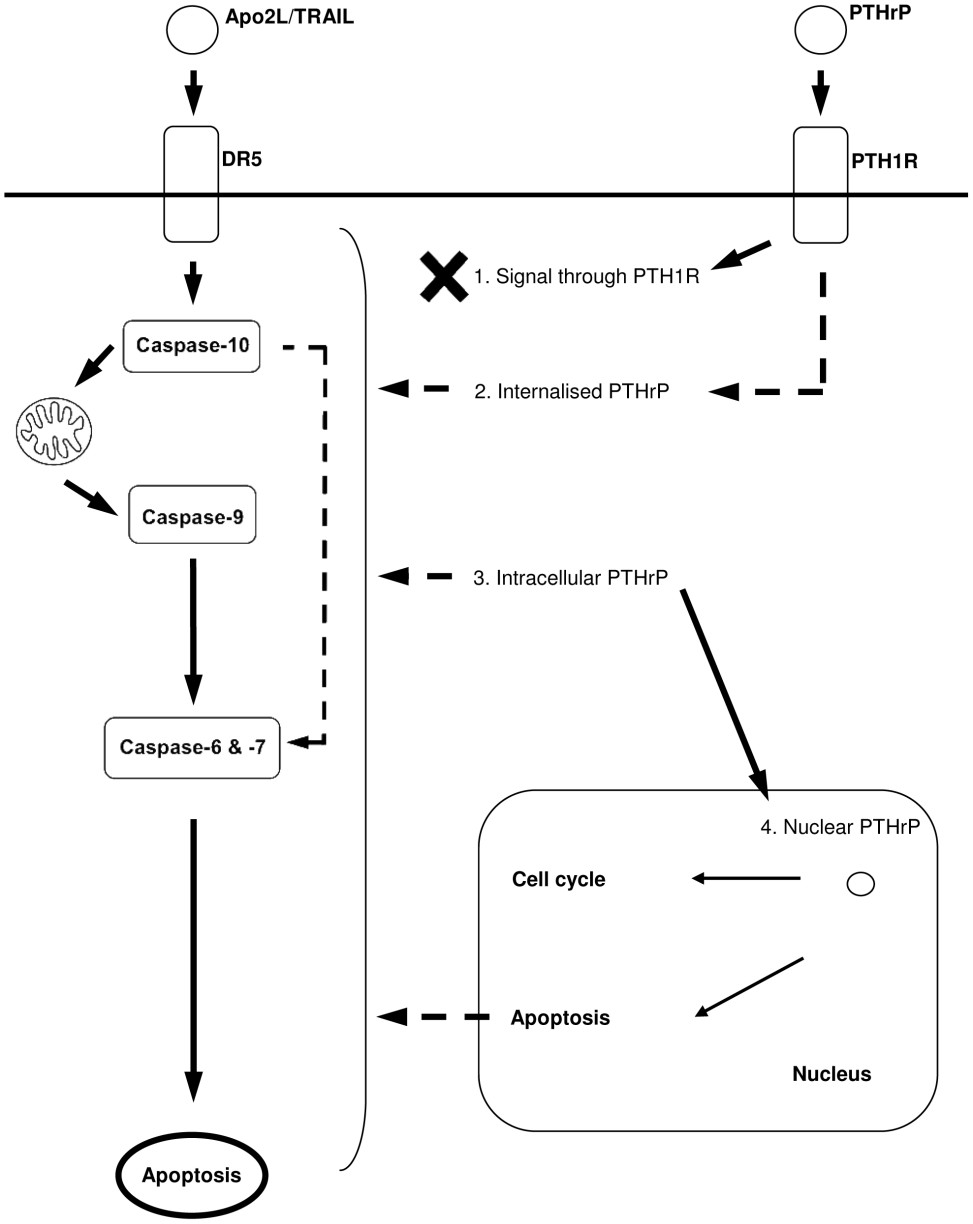
Schematic Diagram of Proposed Model of Relationship Between PTHrP and Apo2L/TRAIL in Breast Cancer Cells. Depicted is a model for PTHrP overexpression and Apo2L/TRAIL induced apoptosis in breast cancer cells. In PTHrP cells preferential binding of Apo2L/TRAIL ligand to TRAIL-R2 drives the activation of caspase-10. This leads to the activation of caspase-9, 6 and 7 and resulting in apoptosis. The actions of PTHrP were not through PTH1R signalling (1), but possibly through actions of internalised PTHrP (2), or intracellular PTHrP (3) or nuclear PTHrP (4): internalised PTHrP via PTH1R (2) is unlikely since exogenous treatment of cells with PTHrP do not result in apoptosis. Nuclear PTHrP can affect cell cycle and caspases to regulate apoptosis. Dotted line indicates indirect and/or unknown interaction and solid lines indicates direct interaction.

Results from this study provide further evidence of a role for PTHrP in apoptosis, whereby PTHrP overexpression sensitised MCF-7 breast cancer cells to Apo2L/TRAIL-induced apoptosis. The actions of PTHrP to confer this phenotype were the result of intracellular signalling of PTHrP and not through its extracellular actions. Apo2L/TRAIL-induced apoptosis of PTHrP overexpressing cells occurred through binding of Apo2L/TRAIL to TRAIL-R2, resulting in the activation of caspase-10 and -9, which suggests that the intrinsic apoptotic pathway is activated, along with the subsequent activation of the effector caspases, caspase-6 and -7, leading to apoptosis (**Fig.** **7**).

Apo2L/TRAIL targeted therapies such as Apo2L/TRAIL peptide and receptor agonists are currently evaluated in clinical trials in a variety of tumours. Apo2L/TRAIL has proven to be a prospective molecule because of its potent ability to induce death, selectivity to tumour cells and lack of significant toxicity in normal organs [12]. This was demonstrated *in vivo*, whereby SCID mice with subcutaneously implanted human tumours displayed a decrease in tumour size when treated with Apo2L/TRAIL, and no toxic effects were observed in normal tissues [36]. In a mouse model where MDA-MB-231 cells were transplanted into the tibiae of athymic nude mice, it was shown that treatment with Apo2L/TRAIL reduced osteolysis and tumour burden and no detectable soft tissue invasion [37]. The same group also demonstrated a similar outcome with Apo2L/TRAIL treatment in a mouse model of multiply myeloma [38]. However, one of the limitations of Apo2L/TRAIL therapy is resistance of tumours to its treatment, in particular breast cancer [39], [40]. Studies have demonstrated that combination treatment using various agents with Apo2L/TRAIL may overcome Apo2L/TRAIL resistance. Treatment with the histone deacetylase inhibitor, suberoylanilide hydroxamic acid (SAHA), can sensitise generated Apo2L/TRAIL-resistant MDA-MB-231 cells to Apo2L/TRAIL induced apoptosis [41]. Consistently, Apo2L/TRAIL resistant explanted breast tumour cells were re-sensitised when Apo2L/TRAIL was used in combination with chemotherapeutic drugs including taxol, etoposide, doxorubicin, cisplatin or SAHA [37]: many of these agents are known to enhance PTHrP expression [23].

One of the proposed mechanisms of Apo2L/TRAIL sensitivity in cancer cells is dependent on the level of expression of the Apo2L/TRAIL receptors. Results from these PTHrP overexpression studies showed an increase in cell surface expression of the death receptors TRAIL-R1 and TRAIL-R2, which may explain in part the cells susceptibility to exogenous Apo2L/TRAIL-induced apoptosis. Consistent with this, previous studies have demonstrated that increased cell surface expression of Apo2L/TRAIL death receptors sensitise tumour cells to Apo2L/TRAIL. Higher cell surface expression levels of TRAIL-R2 was shown to be associated with the preferential response of ovarian, colon and renal cancer cells lines to TRAIL-R2 agonistic antibodies [42]–[45]. However, Apo2L/TRAIL apoptotic signalling via its death receptors may not be equivalent or interchangeable in different tumours and settings. Several studies have demonstrated that membrane expression of Apo2L/TRAIL death receptors does not associate with the cell’s susceptibility toward either TRAIL-R1 or –R2 stimulation [46]–[48]. For example, cancer cell lines, including lung, colon and breast, all expressing similar membrane levels of TRAIL-R1 and –R2 expression, displayed a higher sensitivity to TRAIL-R2 signalling [46]. In addition, knock-down of TRAIL-R2 in MDA-MB-231 inhibited the toxic effects of Apo2L/TRAIL, while loss of TRAIL-R1 had no effect [40]. Preferential binding and signalling with TRAIL-R2 was also demonstrated in this study as the Apo2L/TRAIL-induced apoptotic response was inhibited when PTHrP overexpressing MCF-7 cells were treated with TRAIL-R2 antagonistic antibodies.

Alternatively, PTHrP may alter a cells susceptibility to apoptosis by altering the ratio of anti- and pro-apoptotic factors, including members of the Bcl-2 family. PTHrP has been demonstrated to induce the up-regulation of Bcl-xS, Bad, Rip1 and switches on the expression of caspases in MDA-MB-231 cells [31]. The ratios of the apoptosis regulating proteins Bcl-2 to Bax and Bcl-X_L_ to Bax were higher in breast cancer cells overexpressing PTHrP but not in NLS-mutated PTHrP overexpressing cells [19]. Additionally, mitogenic effects of PTHrP have been attributed to intracrine actions, as mutation of the NLS region of PTHrP ablated the apoptotic and proliferative response and exogenous PTHrP had no effect [29]. In contrast, studies have demonstrated that intracrine PTHrP protects against serum starvation induced apoptosis in MCF-7 cells [19] and that nuclear localisation of PTHrP in chondrocytes delayed apoptosis induced by serum starvation [49]. Results from this study further support an intracellular role of PTHrP, as treatment with various fragments of PTHrP did not affect Apo2L/TRAIL induced apoptosis.

Results described herein demonstrate that PTHrP overexpression sensitises breast cancer cells to Apo2L/TRAIL-induced apoptosis. Thus, clinical implications from this study suggest that PTHrP may be an indicative diagnostic factor in determining therapeutic strategies with Apo2L/TRAIL in treating cancer patients. Currently, serum levels of patients with HHM are assessed using RIA to detect PTHrP, this assessment would be a potential factor in determining Apo2L/TRAIL sensitivity of patients with PTHrP positive tumours. However, new treatment strategies are of importance not only for primary breast tumours but also for patients with metastatic disease. PTHrP is normally expressed in the majority of breast cancers, especially in the late stage metastatic breast cancers, and as such, the expression of PTHrP by cancers may be influential in determining the effectiveness of Apo2L/TRAIL-based therapies in a clinical setting.
